# Single-Step Purification of Catalase Enzyme From Human Blood Erythrocytes Using Affinity Chromatography Technique

**DOI:** 10.1155/2024/2222098

**Published:** 2024-07-09

**Authors:** Kübra Çıkrıkcı, Nahit Gencer

**Affiliations:** Department of Chemistry Faculty of Arts and Sciences Balikesir University, Balikesir, Türkiye

**Keywords:** affinity chromatography, catalase, human blood erythrocytes, purification

## Abstract

In this study, we aimed to isolate and purify catalase from human blood erythrocytes by using a newly synthesized affinity gel. The synthesized *ω*-amino hexyl agarose-1,2,3-triazole-5-carboxylic acid affinity gel was analyzed by FT-IR. Then, different buffer, pH, and ionic strength parameters were optimized to determine the equilibration, washing, and elution buffer conditions. The catalase was purified from human blood erythrocytes with a specific activity of 45.58 EU/mg, purification fold of 529.50, and a yield of 0.416% using the synthesized new affinity gel. The purity and molecular weight of the enzyme were analyzed by SDS-PAGE, and a single band at 60 kDa was observed for catalase. The optimum reaction temperature of the catalase was found to be 30°C, while the thermal stability temperature was 60°C. The Km and Vmax of the enzyme for hydrogen peroxide were calculated at 0.125 mM and 2500 U mL^−1^, respectively.

## 1. Introduction

Enzymes are environmentally friendly biocatalysts with high efficiency that are used in industrial fields for sustainable production. As the demand for enzymes increases in industrial fields, their approach to purification is expected to improve [1]. Enzymes can be purified using many different chromatographic purification methods. However, in industrial areas, one-step purification of enzymes from raw sources is very important to reduce production costs [2, 3]. Catalase is widely used in industry for H_2_O_2_ removal, oxidation prevention, biosensors, and bioremediation [4–7]. Catalase is also widely used in clinical studies as it is an antioxidant enzyme, and catalase deficiency has been associated with several diseases, such as acatalasemia, vitiligo, coronary artery disease, cancer, and type 2 diabetes [8–12]. Catalase (EC 1.11.1.6), an antioxidant enzyme located in the peroxisomes of nearly all aerobic microorganisms, plants, and animals, protects cells from ROS by reducing hydrogen peroxide to water and oxygen. Catalase activity is high in mammalian liver and erythrocytes and low in tissues such as the heart and brain. Human erythrocyte catalase (HEC) is a 244 kDa tetrameric protein with more than 500 amino acid residues in each molecule, each containing four identical subunits of 59.7 kDa bound to its active site, four heme groups, and four NADPHs [13]. In addition, it has been reported that cyanide, azide, and fluoride cause reversible inhibition of catalase, while 3-amino-1,2,4-triazole (3-AT) causes irreversible inhibition [14]. Affinity chromatography is a practical alternative for one-step purification of enzymes based on the principle of specific immobilization of a biological ligand (for example, substrate, coenzyme, hormone, and inhibitor) on an insoluble support material (matrix) [15]. The selectivity and simplicity of this method are exploited for the purification of many biomolecules, biopharmaceuticals, and other agents [2].

In this study, a novel affinity gel was synthesized and optimized. Then, catalase was purified from human blood erythrocytes. In the literature, catalase has been purified by different chromatography techniques and from different sources, but studies on catalase purification from human blood erythrocytes are limited. In addition, the affinity chromatography technique, a type of adsorption chromatography, and 3-AT-5-carboxylic acid ligand were used in this study for the first time.

## 2. Materials and Methods

### 2.1. Chemicals

*ω*-Aminohexyl agarose, hydrogen peroxide (H_2_O_2_), dipotassium phosphate (K_2_HPO_4_), potassium dihydrogen phosphate (KH_2_PO_4_), Tris-HCl, N,N,N',N'-tetramethylenediamine (TEMED), tri-hydroxymethylaminomethane (Tris-Base), 3-AT-5-carboxylic acid, and 1-ethyl-3-(3-dimethylaminopropyl) carbodiimide hydrochloride (EDC) were obtained from Sigma Chemical Co. Prestained Protein Ladder, “PageRuler™ Plus” 10–180 kDa, was purchased from Thermo Fisher Scientific. All other chemical materials used are of analytical purity. Human blood samples were obtained from Balikesir University Faculty of Medicine. The study was approved by the local Institutional Ethics Committee, and informed consent was obtained from all participants.

### 2.2. Methods

#### 2.2.1. Synthesis of Affinity Gel for Catalase Purification

Twenty microliters of *ω*-aminohexyl agarose was prepared by washing with 60 mL of distilled water. This procedure was repeated three times. Thirty milligrams of 3-AT-5-carboxylic acid (ligand) was dissolved in 40 mL of distilled water. Then, 100 *μ*L of 1-ethyl-3-(3-dimethylaminopropyl) carbodiimide hydrochloride (EDC) crosslinking reagent was added to the ligand solution. The prepared ligand solution was rapidly added to the matrix solution. The pH was kept constant at 4.5 for the reaction to take place, and the solution was incubated at 25°C for 18 h. The gel solution was washed with 1 L of distilled water and 250 mL of NaOH (pH: 4.5) buffer by transferring the Buchner funnel. The synthesized *ω*-aminohexyl agarose-3-AT-5-carboxylic acid affinity gel was analyzed by FT-IR. This analysis was performed with a Perkin Elmer Spectrum 100 device in the wavelength range of 4000–650 cm^−1^.

#### 2.2.2. Preparation of Hemolysate

Blood sample (10 mL) was collected from healthy volunteers. The blood sample was centrifuged at 5000 rpm for 20 min at 4°C. Plasma and buffy coat were separated and washed twice with 0.9% NaCl. Then, the blood sample was hemolyzed with three times the volume of cold water. The hemolysate was centrifuged again at 15,000 rpm for 40 min at 4°C.

#### 2.2.3. Optimization of Equilibration, Washing, and Elution Buffers of the Affinity Gel

For the optimization of equilibration, washing, and elution buffers of the affinity column, each condition was prepared as described in this section, and 10 mL of hemolysate was used for each. The equilibration buffer was prepared with 25 mM Tris-Base/0.1 M NaCl in different pH values (9.5-9-8.5-8-7.5-7-7-6.5-6-6-5.5-5) [16]. The column was equilibrated with each buffer individually, and the optimum pH was determined to be 8.5. Subsequently, 25 mM Tris-Base (pH: 8.5) was prepared with different concentrations (0.05-0.1-0.15-0.2 M) of NaCl, Na_2_SO_4_, and NaSCN to determine the ionic strength of the equilibration buffer. Then, the wash buffer was prepared with 25 mM Tris-base/22 mM NaCl at the same pH values as the equilibration buffer optimization. However, to determine the ionic strength, 25 mM Tris-Base (pH: 9.5) was prepared with different concentrations (22-24-26-28 mM) of NaCl, Na_2_SO_4_, and NaSCN and the column was washed with these buffers. Finally, 0.15 M Na_2_HPO_4_ (pH:5) was prepared at different pH (9.5–5) and concentrations (20-24-28 mM) with NaCl, Na_2_SO_4_, and NaSCN, respectively, to determine the ionic strength for the elution buffer. Purification was carried out with all the prepared buffer solutions separately, and the highest activity elution condition for the catalase was determined as 0.2 M Na_2_HPO_4_ (pH: 5).

#### 2.2.4. Optimum and Stability Temperature of Catalase

To determine the optimum temperature at which the activity of catalase was purified from human blood erythrocytes, it was measured spectrophotometrically with 10°C of increases ranging from 10°C to 60°C. These measurements were repeated three times, and an activity-temperature plot was generated. The thermal stability of purified catalase was determined by incubating it at various temperatures (4°C, 20°C, 30°C, 40°C, 50°C, and 60°C) for 1 h, with enzyme activity measured spectrophotometrically and recorded every 10 min.

#### 2.2.5. Protein Determination

Quantitative protein determination was performed at 595 nm by the Bradford method [17]. Bovine serum albumin (BSA) was used as a standard, and a purification table was prepared.

#### 2.2.6. SDS-PAGE Analysis

To check the purity of the purified catalase and determine its molecular weight, SDS-PAGE was performed according to the Laemmli method [18].

#### 2.2.7. Determination of Catalase Activity and Kinetic Parameters

The catalase activity was measured spectrophotometrically at 240 nm according to the method of Aebi [19]. One unit (enzyme units [EUs]) of the catalase activity was defined as the amount of enzyme that catalysed the decomposition of 1 *μ*mol H_2_O_2_ (30 mM) at 25°C in phosphate buffer (50 mM, pH 7.0) in 60 s. The molar absorptivity for H_2_O_2_ at 240 nm was assumed to be 40.0 M^−1^ cm^−1^. Enzyme activity was assessed at a wavelength of 240 nm using six different substrate concentrations at pH 7.0 in order to determine Km and Vmax values. Following the measurements, calculations were conducted by plotting the Lineweaver–Burk graph (1/[V]-1/[S]). Each activity measurement was repeated three times and recorded.

## 3. Results

**Figure 1:**
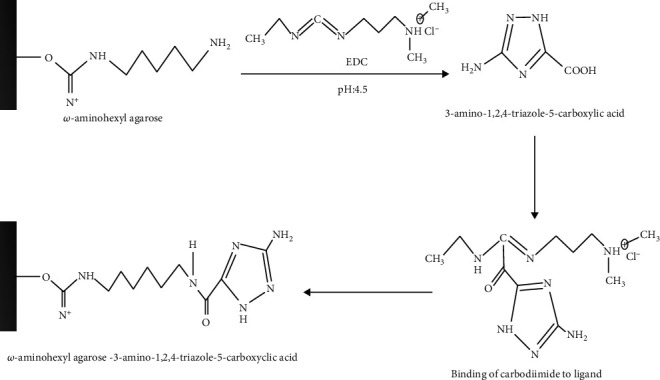
Preparation of the new affinity gel for purification of the CAT enzyme.

**Figure 2:**
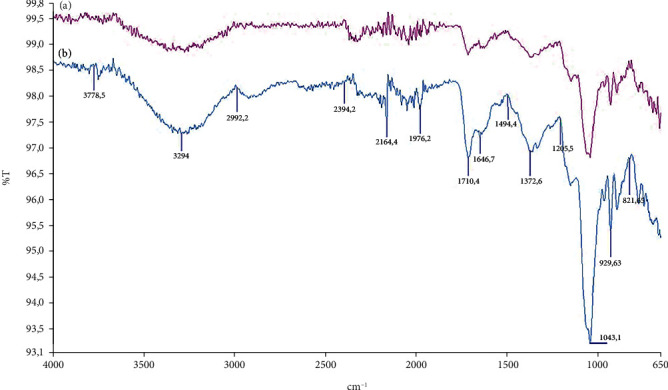
FT-IR analysis of the aminohexyl agarose gel which was used as a matrix and the synthesized affinity gel was evaluated in the wavelength range of 4000-650 cm^−1^. (a) *ω*-Aminohexyl agarose (red diagram). (b) *ω*-Aminohexyl agarose-3-amino-1,2,4-triazole-5-carboxylic acid (blue diagram).

**Figure 3:**
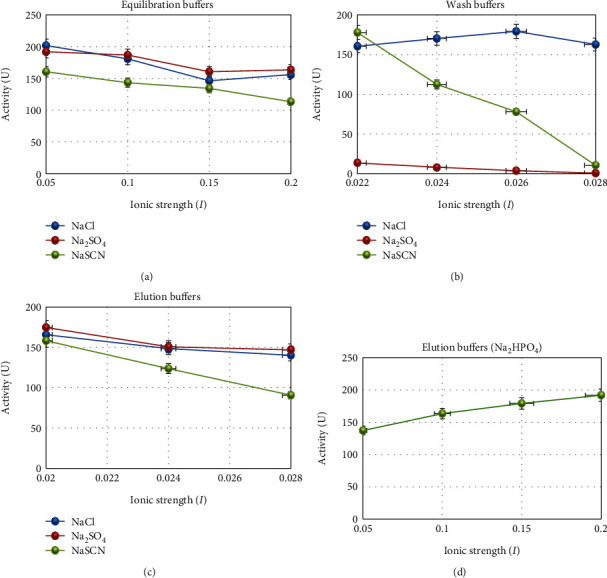
(a) Determination of the ionic strength of the equilibration buffer (25 mM Tris-Base was prepared 0.05-0.1-0.15-0.2 M NaCl, Na_2_SO_4_, and NaSCN, respectively). (b) Determination of the ionic strength of the wash buffer (25 mM Tris-Base was prepared 0.022-0.1-0.024-0.026-0.028 mM NaCl, Na_2_SO_4_, and NaSCN, respectively). (c) Determination of the ionic strength of the elution buffer (25 mM Tris-Base was prepared 0.02-0.024-0.026-0.028 mM NaCl, Na_2_SO_4_, and NaSCN, respectively). (d) Determination of the ionic strength of the elution buffer (Na_2_HPO_4_ at different concentration was prepared as 0.05 M, 0.1 M, 0.15 M, and 0.2 M, respectively).

**Figure 4:**
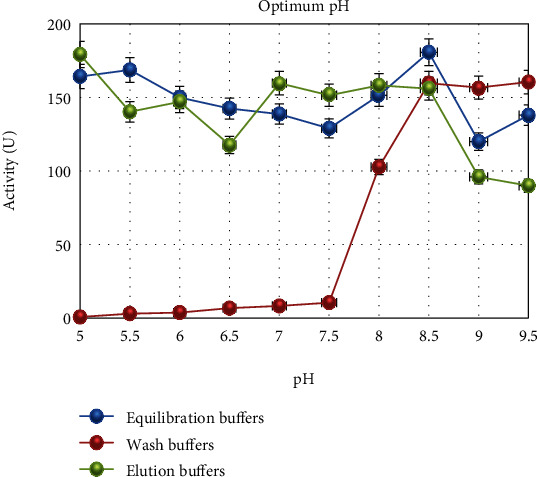
Optimization of equilibration, washing, and elution buffers at different pH ranges (9.5–5).

**Figure 5:**
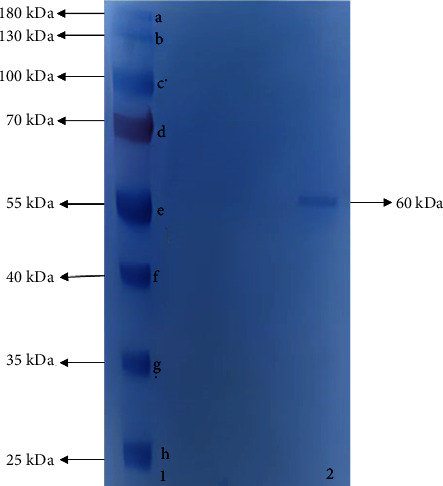
SDS polyacrylamide gel electrophoresis of human blood erythrocyte CAT purified by affinity gel. Line 1: the protein standards ((a) 180 kDa, (b) 130 kDa, (c) 100 kDa, (d) 70 kDa, (e) 55 kDa, (f) 40 kDa, (g) 35 kDa, and (h) 25 kDa). Line 2: human blood erythrocyte CAT.

**Figure 6:**
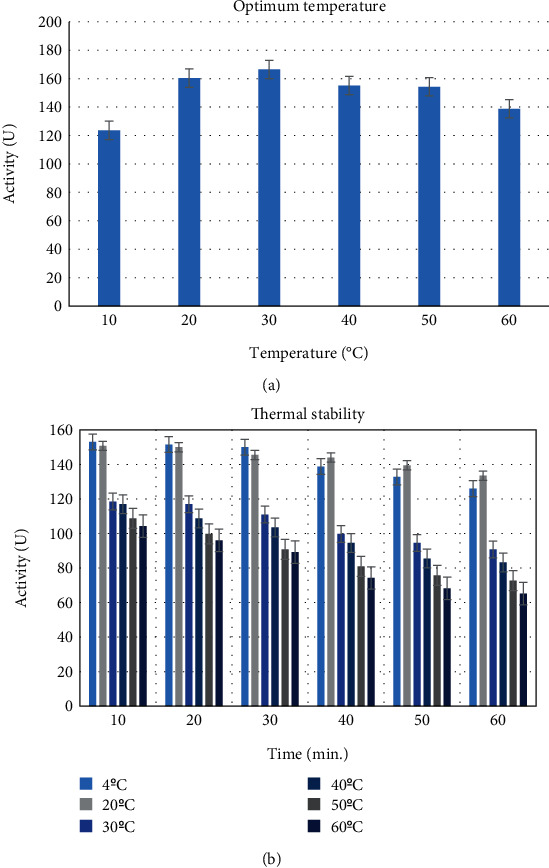
(a) The effect of different temperatures on CAT activity. (b) Thermal stability of CAT at different temperatures.

**Figure 7:**
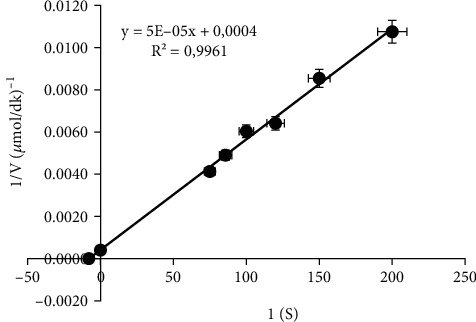
Lineweaver–Burk graph of human blood erythrocyte CAT obtained by using H_2_O_2_ substrate.

In this study, catalase was purified from human blood erythrocytes by affinity gel with *ω*-aminohexyl agarose-3-AT-5-carboxylic acid chemical structure (Figure 1). Commercially purchased *ω*-aminohexyl agarose gel (matrix) eliminates the need for a spacer arm because it contains an amino group (-NH_2_) and has a length of six carbons. The high degree of purification in affinity chromatography gel synthesis depends on the specificity and degree of binding of the selected ligand. For this reason, 3-AT-5-carboxylic acid, a specific inhibitory derivative of the catalase, was preferred as the ligand. In order to activate the amino group (-NH_2_) in the gel structure and the carboxyl group (-COOH) in the ligand structure, affinity gel was synthesized using the carbodiimide (EDC) activation method. Binding of the ligand to the primary amino group of the matrix through the carbodiimide forms an amide bond, and this bond is resistant to chromatographic processes [20, 21]. FT-IR analysis of the aminohexyl agarose gel which was used as a matrix and the synthesized affinity gel was evaluated in the wavelength range of 4000–650 cm^−1^. The IR spectra of the *ω*-aminohexyl agarose gel showed -NH peaks between 3378.5 and 3294 cm^−1^ [22]. Correspondingly, the -NH group shows a peak between 3500 and 3350 cm^−1^. In addition, IR analysis of the primary amide group gives a peak in the spectrum range 1900–1650 cm^−1^ [23]. The primary amide group gave a peak at 1710.4 cm^−1^ in the synthesized affinity gel. A comparison of the FT-IR spectra analysis results is shown in Figure 2. As a result of the affinity gel optimization studies, it was determined that the most appropriate buffer for equilibration was 25 mM Tris-Base/0.05 M NaCl (pH: 8.5) (Figure 3(a)), while the most appropriate buffer for washing was 25 mM Tris-Base/26 mM NaCl (pH: 9.5) (Figure 3(b)). It was determined that the catalase activity was low by ionic strength assays for elution (Figure 3(c)). Subsequently, purification was performed using Na_2_HPO_4_ (pH: 5) buffers prepared at different concentrations (0.05 M, 0.1 M, 0.15 M, and 0.2 M) (Figure 3(d)). It was found that the highest activity for elution buffer was obtained with 0.2 M Na_2_HPO_4_ (pH: 5). The activity variation of equilibration, washing, and elution buffers at different pH values is shown in Figure 4. Quantitative protein determination was performed by the Bradford method for catalase purified from human blood erythrocytes by affinity chromatography. The catalase was purified from human blood erythrocytes with a specific activity of 45.58 EU/mg, purification fold of 529.50, and a yield of 0.416% using *ω*-aminohexyl agarose-3-AT-5-carboxylic acid affinity chromatography (Table 1). To determine enzyme purity and molecular mass, SDS-PAGE was performed, and a single band was obtained at a molecular weight of approximately 60 kDa (Figure 5). The determined molecular mass was similar to that of catalase purified by different chromatographic methods in previous studies. For example, dog erythrocytes at 57.5 kDa [24], sheep erythrocyte at 60.52 kDa [25], and human erythrocytes at 60 kDa [26] were reported. The activity of purified catalase at different temperatures (10-20-30-40-50-60°C) was assayed and determined that the optimum reaction temperature was 30°C (Figure 6(a)). The optimum temperature determined was similar to the catalase purified from different tissues in previous studies. For example, chicken liver at 30°C [25], sheep erythrocyte at 30°C [25], camel liver at 45°C [27], and bovine liver at 40°C [28] were reported. Temperature stability was tested for 1 h by measuring catalase activity every 10 min at different temperatures. The catalase showed a high activity over a wide range of temperatures, from 4°C to 30°C, and showed low activity at 40-50-60°C (Figure 6(b)). The enzyme kinetics of the catalase were measured spectrophotometrically using different H_2_O_2_ concentrations as substrates, and a Lineweaver–Burk plot was used to calculate Km: 0.125 mM, and Vmax: 2500 EU/mL (Figure 7).

## 4. Discussion

Affinity chromatography is a protein purification technique based on the specific immobilization of a biological ligand (substrate, coenzyme, hormone, antibody, nucleic acid, inhibitor, etc.) on an insoluble support material (matrix) [15]. Affinity gel synthesis consists of several steps, including immobilization of the ligand to the matrix, removal of unwanted impurities, and pure elution of the target protein. Various gels such as Sephadex, Sepharose, and Biogel are used as the matrix. The functional groups of the carrier matrix (-S, -SH, -OH, -NH_3_, etc.) are very important for the covalent binding of the groups in the side chains of the amino acids in the enzyme structure [29, 30]. The matrix and ligand must be activated with reagents such as cyanogen bromide, carbodiimide, and glutaraldehyde for this binding to occur. After the ligand immobilization is performed to the activated matrix, the molecule to be purified is bound to the ligand on the column. The molecule retained in the column is eluted with a buffer solution containing a substance of higher affinity than the ligand, and hundreds of fold purification are achieved in a single step [31, 32].

In this study, catalase was purified from human blood erythrocytes by one-step *ω*-aminohexyl agarose-3-AT-5-carboxylic acid affinity chromatography with 529.60-fold. Catalase, which is found in high amounts in blood erythrocytes, was purified from chicken erythrocytes by acetone precipitation, ethanol–chloroform treatment, CM-cellulose, and Sephadex G-200 chromatography with 136-fold in a previous study [33]. In another study, catalase was purified from sheep erythrocytes in three steps by DEAE-Sephadex A50 ion exchange chromatography method with 643.90-fold [25]. Catalase studies purified from human blood erythrocytes, on the other hand, are limited in the literature. Catalase from human blood erythrocytes was purified by a group of scientists with copper-chelate affinity chromatography technique in two steps with 565-fold [26]. The affinity gel that we synthesized offers the possibility to purify catalase in one step compared to other reported studies. In addition, a single band at approximately 60 kDa was observed by SDS-PAGE, and our results are in agreement with these studies.

## 5. Conclusion

In the current study, a novel affinity gel was synthesized for the first time, utilizing 3-AT-5-carboxylic acid, a specific inhibitory derivative of catalase, as a ligand. Catalase purification from human blood erythrocytes was achieved via one-step affinity chromatography. Given the diverse industrial applications of catalase, the development of fast, practical, and applicable chromatographic methods for its purification holds significant importance. The newly synthesized affinity gel presented in this study may offer an efficient approach for catalase purification from novel sources.

## Figures and Tables

**Table 1:** Summary of purification procedure for human blood erythrocyte catalase by a *ω*-aminohexyl agarose-3-amino-1,2,4-triazole-5-carboxylic acid affinity column chromatography.

**Purification step**	**Total volume (mL)**	**Activity (EU/mL)**	**Total activity (EU)**	**Protein (mg/mL)**	**Total protein (mg)**	**Specific activity (EU/mg)**	**% yield**	**Purification fold**
Hemolysate	10	134.25	1342.5	1559.60	15596	0.086	100	—
Catalase	3	186	558	4.081	12.242	45.579	0.416	529.50

## Data Availability

No underlying data was collected or produced in this study.

## Nomenclature

3-AT: 3-Amino-1,2,4-triazole
BSA: Bovine serum albumin
CAT: Catalase
EDC: 1-Ethyl-3-(3-dimethylaminopropyl) carbodiimide hydrochloride
ROS: Reactive oxygen species
